# Humidity sensing using Zn_(1.6 − x)_Na_0.4_Cu_x_TiO_4_ spinel nanostructures

**DOI:** 10.1038/s41598-023-50888-6

**Published:** 2024-01-04

**Authors:** A. M. Mansour, Mohamed Morsy, Amany M. El Nahrawy, Ali B. Abou Hammad

**Affiliations:** 1 Solid State Physics Department, Physics Research Institute, National Research Centre (NRC), 33 El-Bohouth St., Dokki, Cairo 12622 Egypt; 2 Building Physics and Environment Institute, Housing and Building National Research Center (HBRC), Dokki, Giza, 12311 Egypt; 3 Nanotechnology Research Centre (NTRC), The British University in Egypt (BUE), Suez Desert Road, El-Sherouk City, Cairo 11837 Egypt

**Keywords:** Materials science, Physics

## Abstract

In this paper, we present a humidity sensing material based on nanostructured Zn_(1.6 − x)_Na_0.4_Cu_x_TiO_4_ spinel to enhance optical and sensitivity performance. Nano-porous of Zn _(1.6 − x)_ Na_0.4_Cu_x_TiO_4_ spinel were synthesized using sol gel reactions and calcined at 700 °C. The nanostructures of Zn_(1.6 − x)_Na_0.4_Cu_x_TiO_4_ spinel underwent thorough characterization through multiple techniques. X-ray diffractometry (XRD) coupled with Rietveld refinement using FullProf software, transmission electron microscopy (TEM), Raman Spectroscopy, and optical analysis were employed to assess various aspects of the nanostructures. These techniques were utilized to determine the phase composition, particle size distribution, chemical bonding, and the tunable band gap of the nanostructures. The X-ray diffraction (XRD) analysis of Zn_(1.6 − x)_Na_0.4_Cu_x_TiO_4_ samples revealed well-defined and prominent peaks, indicating a highly crystalline cubic spinel structure. The lattice parameter was decreased from 8.4401 to 8.4212 Å with increasing Cu content from 0 to 1.2 mol%. UV–visible diffuse reflectance spectra were employed to investigate the optical characteristics of copper-doped Zn_1.6_Na_0.4_TiO_4_. The applicability of Cu@NaZT spinel nanostructures in humidity sensors was evaluated at ambient conditions. The fabricated sensor was investigated in a wide span of humidity (11–97%). The examined sensor demonstrates a low hysteresis, excellent repeatability, fast response and recovery. The response and recovery times were estimated to be 20 s and 6 s respectively. The highest sensitivity was achieved at 200 Hz. The proposed sensor can be coupled easily with electronic devices as the humidity–impedance relationship is linear.

## Introduction

The extensive discourse on humidity sensors arises from their wide-ranging potential applications, besides their well-established uses in agricultural monitoring, industry, medical applications, and weather prediction, their relevance is expanding into new areas, including their utilization in intelligent domestic settings for purposes like patient detection^1–3^. Humidity sensors are often used in industries where humidity levels must be monitored due to their possible effects on manufacturing processes and products. These sensors must possess high sensitivity that can detect changes in the environment’s humidity level^4,5^. Among the above-mentioned applications of humidity sensors, some applications related to human beings are proposed. These applications comprise respiration breath rates that are related to some diseases, also the thermal comfort of closed areas is related to the amount of relative humidity in such areas^6–10^. Ceramics have attracted considerable interest for their potential application in humidity sensors due to their ability to withstand exacting physical and chemical conditions^1,11,12^. However, most ceramics typically require higher sintering temperatures, often exceeding a thousand degrees Celsius, which can make them incompatible with the integration into IC fabrication processes. Nonetheless, the use of the sol–gel technique offers a solution by eliminating the necessity for these elevated processing temperatures, enabling smooth integration of ceramics into IC fabrication procedures^13,14^. When contrasted with conventional active materials, spinel nanostructures are regarded as promising choices for applications in optoelectronics and humidity sensors. They exhibit wide bandgap, excellent thermal and mechanical stability, and excellent magnetic and electrical properties. These properties make the spinel nanostructures more suitable for various applications extending from sensors to optoelectronic applications^15,16^. This is primarily due to their favorable characteristics in terms of preparation methods and physicochemical properties, which encompass a substantial surface area, elevated conductivity, enhanced carrier mobility, and the ability to control the band gap^16–18^. It's important to highlight those sensors and optoelectronics built upon the AB_2_O_4_ structure of spinel have demonstrated considerable potential and offer clear benefits compared to conventional materials such as TiO_2_ or ZnO^16,19^.

Spinel’s AB_2_O_4_ type have attracted much research interest due to their adaptable practical applications hence the presence of various metal oxides, mixed ferrites and metal oxides have given good sensitivity to some humidity and gases. Also, spinel structures have characteristic with their physical properties such as dielectric constant, electrical conductivity, and the thermoelectric power rise from the excellent ability of these composites to distribute their cations between the tetrahedral-A and the octahedral-B-sites^20–22^. Titanate Perovskites such as ZnTiO_3_ exhibited good light photocatalytic performance and absorption properties because of their low bandgap energy, linked with the commercial semiconductors, and helped the charge carrier movement in the interfaces^23–25^.

These advantages encompass multifunctionality, superior recyclability, and increased stability.

Additionally, extensive documentation and verification have established that composite sensors, optoelectronics, and catalysts, characterized by their integrated structures, exert a significant influence on various aspects of sensor performance when compared to their standalone counterparts^17,26^. This influence is attributed to their capacity to adeptly control charge carrier densities, expand absorption thresholds into the visible range of the spectrum, and attain larger bandgaps^27–29^. Based on available reports, there is substantial documentation indicating that the ZnO–TiO_2_ nanocomposite structure can assume three distinct structural configurations: Zn_2_TiO_4_, ZnTiO_3_, and Zn_2_Ti_3_O_8_^30,31^. Among these options, the Zn_2_TiO_4_ spinel has garnered significant attention and emerged as a particularly favored and secure choice in recent times^25,32^. This prominence is attributed to its outstanding attributes and versatile applications, encompassing uses such as a pigment, in sensor technology, photocatalysis, water treatment, and various other sectors^33–35^.

However, given the current pressing environmental concerns, it becomes crucial for researchers to channel their efforts toward advancing the study of Zn_2_TiO_4_. This is imperative in order to position this material as a top priority among photocatalysts, given its potential significance in addressing the ecological challenges of our time. In the case of zinc titanate-based structures, an increased titanium content is associated with elevated costs for zinc titanate materials. The cubic structure Zn_2_TiO_4_, consisting of octahedral [TiO_6_] and tetrahedral [ZnO_4_] units, emerges as a potentially superior choice for a sensing material compared to ZnO or TiO_2_, primarily due to its enhanced stability and cost-effectiveness^36–38^.

The complex porous structure of Zn_2_TiO_4_ accommodates active components and facilitates controlled band gaps. It also ensures rapid response times and delivers heightened sensitivity, all of which are pivotal factors in improving the sensing capabilities of humidity sensors^29,34,39,40^.

The physical properties of Zn_2_TiO_4_ can be enhanced by doping with specific metal ions because of their ability to facilitate electron transfer, adjust the band gap, and lower the energy level within the bandgap. CuO, a semiconducting metal oxide, exhibits numerous distinctive physical and chemical characteristics. Notably, it is non-toxic, easily preparable, relatively stable in its properties, and cost-effective in production. Among various copper-based metal oxides, CuO stands out as the preferred material for applications in humidity sensing^41^. Out of the various metallic dopant elements, copper (Cu) has been demonstrated as an effective dopant for enhancing sensitivity^42–44^. Copper oxide (CuO) possesses a significant oxygen adsorption capability on its surface and can be utilized in the fabrication of humidity sensors^4,44^. NaZnTiO_4_-based spinel nanostructures are used in various applications due to their nano size, semiconducting nature, and the progressive properties compared with others^45^. Doping ZnTiO_3_ oxide with sodium oxide (Na_2_O) or other dopants can be done for various reasons, including altering its electronic structure, improving its photocatalytic activity, or enhancing its stability^46,47^.

Doping can modify the electronic band structure and improving its ability to absorb light and generate charge carriers of ZnTiO_3_^25,30,32,47–49^.

Combining Zn_1.6_Na_0.4_TiO_4_ with CuO as a dopant to create affordable humidity sensors with rapid response, high sensitivity, and robust reliability is intriguing. This is because the modification of Zn_2_TiO_4_ by metal oxides can significantly enhance both sensing capabilities and physicochemical properties. Metal oxides can be effectively integrated into the Zn_1.6_Na_0.4_TiO_4_ structure during the sol gel formation, leading to rapid response times and increased stability^25,40,44^. It's essential to emphasize that the synthesis method plays a critical role in shaping the optoelectronic and sensing properties of Zn_1.6_Na_0.4_TiO_4_/CuO nanostructures. One viable approach is the sol–gel method. The sol–gel technique surpasses others in popularity and industrial usage. Its distinctive properties enable the large-scale production of uniformly sized high-quality nanoparticles. Notably, the sol–gel method has the capability to simultaneously produce multiple types of nanoparticles, allowing for the synthesis of alloy products in a single step by combining different metal (or metal oxide) precursors in specific proportions^50–52^. Moreover, the sol–gel technique facilitates the creation of exceptionally homogeneous structures characterized by high purity, nano-porosity, and a large specific surface area. This method also allows for processing at lower temperatures, enabling the production of metal and ceramic nanomaterials within a reduced temperature range^53–55^. The final products in the sol–gel process result from a series of irreversible chemical reactions. Spinel ceramics produced using sol–gel nanoparticles can be attained at low temperatures, facilitated by the influence of small-sized nanoparticles. Herein, Zn_1.6_Na_0.4_TiO_4_ nanoparticles were effectively prepared through the sol–gel technique at 700 °C, exhibiting a cubic structure (Fd-3 m). Additionally, successful incorporation of Cu within the spinel structure was achieved.

In this study, we produced innovative humidity sensor materials in the form of Zn_1.6_Na_0.4_TiO_4_ and Cu-doped spinel nanostructures. This was achieved through a precisely controlled sol–gel reactions, followed by a calcination process at 700 °C. The spinel nanostructures, phase evaluation, morphology, and spectroscopy properties of Zn_(1.6 − x)_Na_0.4_Cu_x_TiO_4_ were comprehensively analyzed through techniques such as X-ray diffraction (XRD) combing with Rietveld refinement software, transmission electron microscopy (TEM), Raman spectroscopy, and ultraviolet–visible (UV–vis) spectroscopy. The introduction of Cu cations into the Zn_1.6_Na_0.4_TiO_4_ nanostructure resulted in internal rearrangements within the Cu@Zn_1.6_Na_0.4_TiO_4_ network structure, which was verified through Rietveld refinement data, TEM and Raman spectroscopy. A fast respond humidity sensor was produced by applying ZnNa_0.4_Cu_0.6_TiO_4_ spinel nanostructures onto FTO coated glass substrate. The humidity sensing performance of ZnNa_0.4_Cu_0.6_TiO_4_ spinel nanostructure was evaluated across a broad range of relative humidity (11–97%) under the effect of 1 VAC. The findings revealed that the ZnNa_0.4_Cu_0.6_TiO_4_ sensor exhibited a rapid response time of 20 s and a fast recovery time of 6 s.

## Expreimental work

### Formation of Zn_(1.6 − x)_Na_0.4_Cu_x_TiO_4_ spinel nanostructures

The Zn_(1.6 − x)_Na_0.4_Cu_x_TiO_4_ ()(x = 0.1, 0.3, 0.6 mol%) spinel nanostructures were prepared by sol gel reactions using reagent grades powders and solvents of sodium acetate trihydrate (CH_3_COONa.3H_2_O), titanium isopropoxide (Ti(OCH(CH_3_)_2_)_4_, zinc acetate Zn(CH_3_COO)_2_.2H_2_O, Copper(II) nitrate trihydrate (Cu(NO_3_)_2_·3H_2_O) (Sigma Aldrich; purity > 99.2%) and diethanolamine (DEA) as stabilizer. Zinc acetate precursors (5.5 g) is dissolved in 50 ml of Ethanol/1.5 g of citric acid and 20 ml H_2_O to form zinc acetate solution before mixed with sodium acetate trihydrate dissolved in the solvent. The initiation of the sol–gel process involved dissolving specified amounts of sodium acetate, zinc acetate, and copper nitrate in distilled water/citric acid (CH_3_COOH). Citric acid served as the primary chelating agent, chosen for its widely recognized ability to enhance the gelation process by virtue of the robust coordination between metal ions and citrate groups. Following this, titanium isopropoxide was directly incorporated into AcAc before being mixed with the earlier dissolved raw materials in their individual solvents. These resulting solutions were subjected to vigorous magnetic stirring for 90 min to ensure complete dissolution. The obtained solution was stirred and heated stirred at temperature between 70 °C in an oven to get the dry precursors.

**Figure 1:**
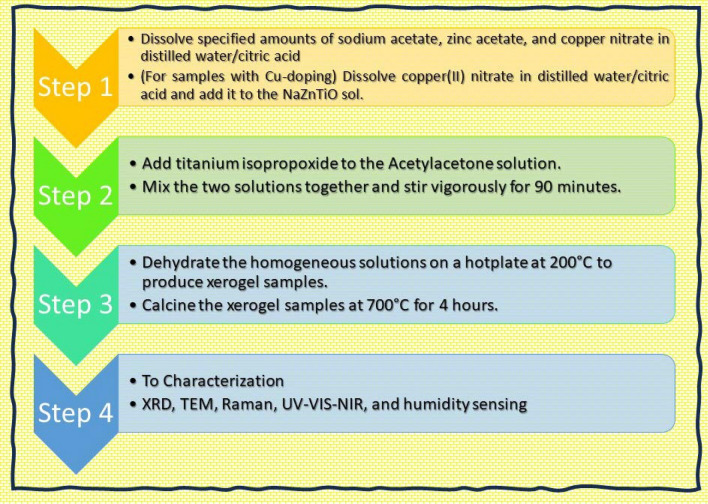
Schematic for the synthesis steps for Zn_(1.6 − x)_Na_0.4_Cu_x_TiO_4_ spinel nanostructures.

For samples containing Cu-doping, copper(II) nitrate was dissolved in a solution of distilled water and citric acid before being introduced into the NaZnTiO4 (NaZT) sol. The homogeneous solutions thus formed by evaporating the solvent, underwent a dehydration process on a hotplate at 200 °C, resulting in the production of xerogel samples as given the Fig. 1. Finally, the resulted powders were calcined to remove the residual solvent and dried. These xerogel samples were subsequently subjected to calcination at 700 °C for a duration of 4 h.

### Characterization

The crystalline phase of the Zn_(1.6 − x)_Na_0.4_Cu_x_TiO_4_ spinel nanostructures was characterized using X-ray (Bruker instrument-D8; XRD) advance diffractometer (Japan) by a monochromatized (CuKα) radiation of wavelength (λ = 1.54056 Å) worked at (40 kV and 40 mA), followed by using Rietveld refinement software to perform structural refinement.

The particles size is estimated using a transmission electron microscope (TEM, JEOL-2100; 200 kV analytical electron microscope; Japan). The particle size distribution of TEM is calculated using (image J Software).

Raman spectra were approved out with a Bruker-instrument (RFS100) using the (1064 nm) excitation line of an (Nd:YAG laser) in the range of 200–1000 cm^−1^.

The JASCO V-570-UV-VIS-NIR spectrophotometer was employed to investigate the optical properties of the prepared powder across a wavelength range of 200–2500 nm using diffuse reflectance unit.

### Fabrication of ZnNa_0.4_Cu_0.6_TiO_4_ spinel sensor

**Figure 2:**
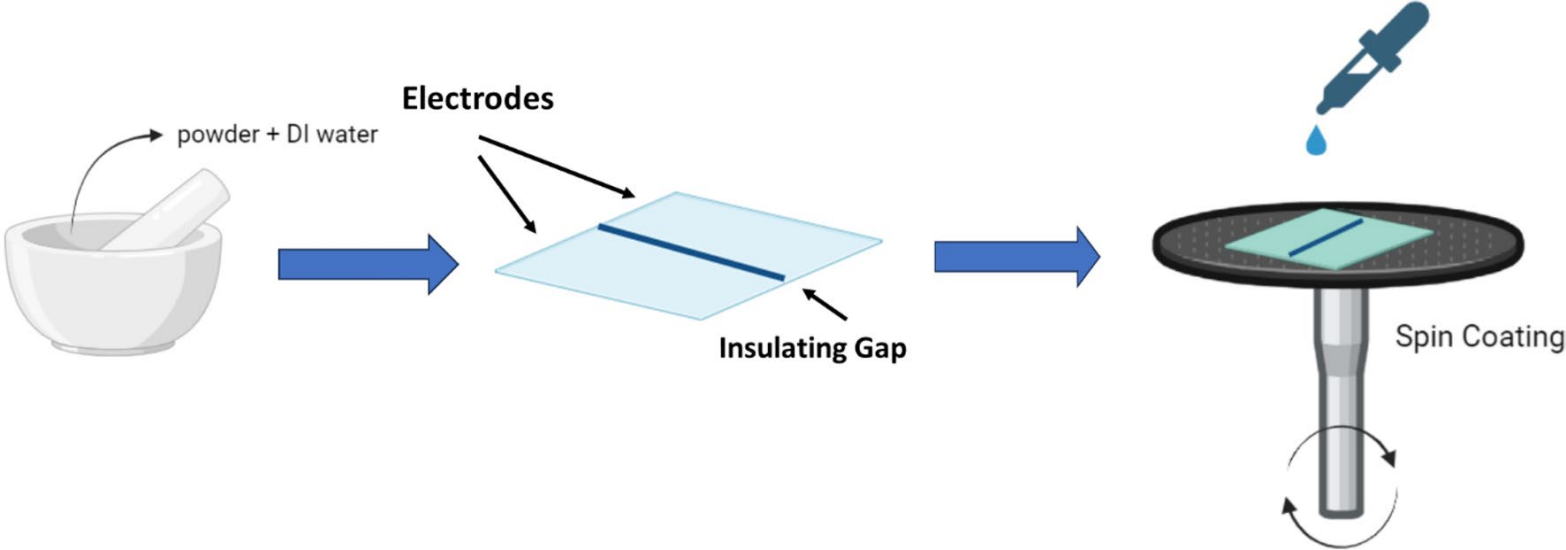
The schematic diagram of the sensor’s fabrication process.

A glass substrate coated with fluorinated tin oxide (FTO) has been utilized as a scaffold for depositing the sensing material. The fabricated ZnNa_0.4_Cu_0.6_TiO_4_ sample was used as humidity sensor. Initially, a gap of 1.5 mm has been created to get two FTO electrodes separated by insulating area. Then the FTO coated glass substrate was cleaned repeatedly with Isopropanol, acetone, and deionized water, then flushed with dry nitrogen. 30 mg of the sensing materials was mixed carefully with 30 µl of deionized water and grinded gently in agate mortar continuously for 5 min to form past. The obtained paste was spread over the FTO substrate via a spin coater instrument operated at 500 rpm for 30 s. The sensing material was allowed to fill the insulated area (1.5 mm gap) between tow FTO electrodes. The fabricated sensor was backed for 1 h at 100 °C to enhance the adhesion of the sensing with the surface of the substrate. The fabricated sensor was subjected to varying levels of relative humidity under the influence of alternating current to promote its stability and reduce signal-to-noise ratio. The schematic diagram of the sensor’s fabrication process is illustrated in Fig. 2.

## Results and discussion

### Crystalline phase study using Rietveld refinement (XRD)

**Figure 3:**
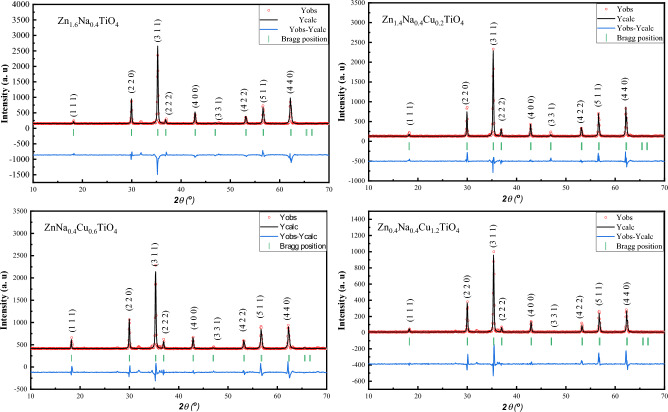
The X-ray diffraction (XRD) data analysis using Rietveld refinement results for Zn_(1.6 − x)_Na_0.4_Cu_x_TiO_4_ (x = 0, 0.2, 0.6, and 1.2) ceramics calcinated at both 700 °C.

Figure 3 displays the X-ray diffraction patterns and the corresponding Rietveld refinement outcomes for the Zn_(1.6 − x)_Na_0.4_Cu_x_TiO_4_ system, where x values range from 0 to 1.2, inclusive. The XRD of Zn_(1.6 − x)_Na_0.4_Cu_x_TiO_4_ shows well-defined and significant peaks, that prove the good crystallinity of the fabricated samples. The well-defined peaks were indexed to the spinel structure Zn_2_TiO_4_ which is consistent with JCPDS # 96-901-2445 of Zn_2_TiO_4_. The absence of spurious diffraction peaks further supports the claim that the synthesized material is singular and in a pure phase.

The Rietveld refinement utilizing FullProf software was performed to explore the structural phase of the fabricated material. The obtained refinement data were recorded in Table 1. As illustrated in Fig. 3, there is a remarkable alignment between the measured XRD profiles and the fitted profiles. The refinement data derived from the Rietveld refinement process confirm the accurate identification of both the sample phase(s) and the crystallographic structure of Zn_(1.6 − x)_Na_0.4_Cu_x_TiO_4_. According to the refinement data in Table 1, the fabricated samples have a face-centered cubic structure (Fd-3 m). The lattice parameter was decreased with the introducing of Cu ions into the nanostructure, which can be attributed to the ionic radius of the Zn ion being greater than the ionic radius of the Cu. Due to the dual tetrahedral and octahedral positions of the spinel structure, spinel material can exhibit various ion occupancies, resulting in the creation of mixed spinel structures. As a result, the arrangement of cations within the tetrahedral or octahedral sites may change. The pure Zn_2_TiO_4_ has fully occupied tetrahedral sites with Zn^2+^ ions, while the octahedral site is shared between the Ti^4+^ and the rest of the Zn^2+^ ions.

**Table 1:** Refinement parameters, reliability factors, lattice parameters, crystallite size (G), molecular weight (Mw), theoretical density, and specific surface area (S_A_) of (Zn_(1.6 − x)_Na_0.4_Cu_x_TiO_4_ (0 ≤ x ≤ 1.2).

Cubic 'F d -3 m	(a)	Cell volume	Crystallite size (G) nm	χ^2^	Rp	Rwp	Rexp	Mw	D_th_ g/cm^3^	S_A_
ZT	8.4401	601.24	56	1.99	5.91	10.7	7.58	225.71	4.9872	21.48
ZT-0.1Cu	8.4397	601.15	62	0.904	4.62	7.73	8.13	225.34	4.9797	19.433
ZT-0.3Cu	8.4284	598.74	57	0.691	1.96	3.98	4.79	224.61	4.9836	21.12
ZT-0.6Cu	8.4212	597.2	59	2.16	27.9	4.8	23.68	223.5057	4.9718	20.454

**Figure 4:**
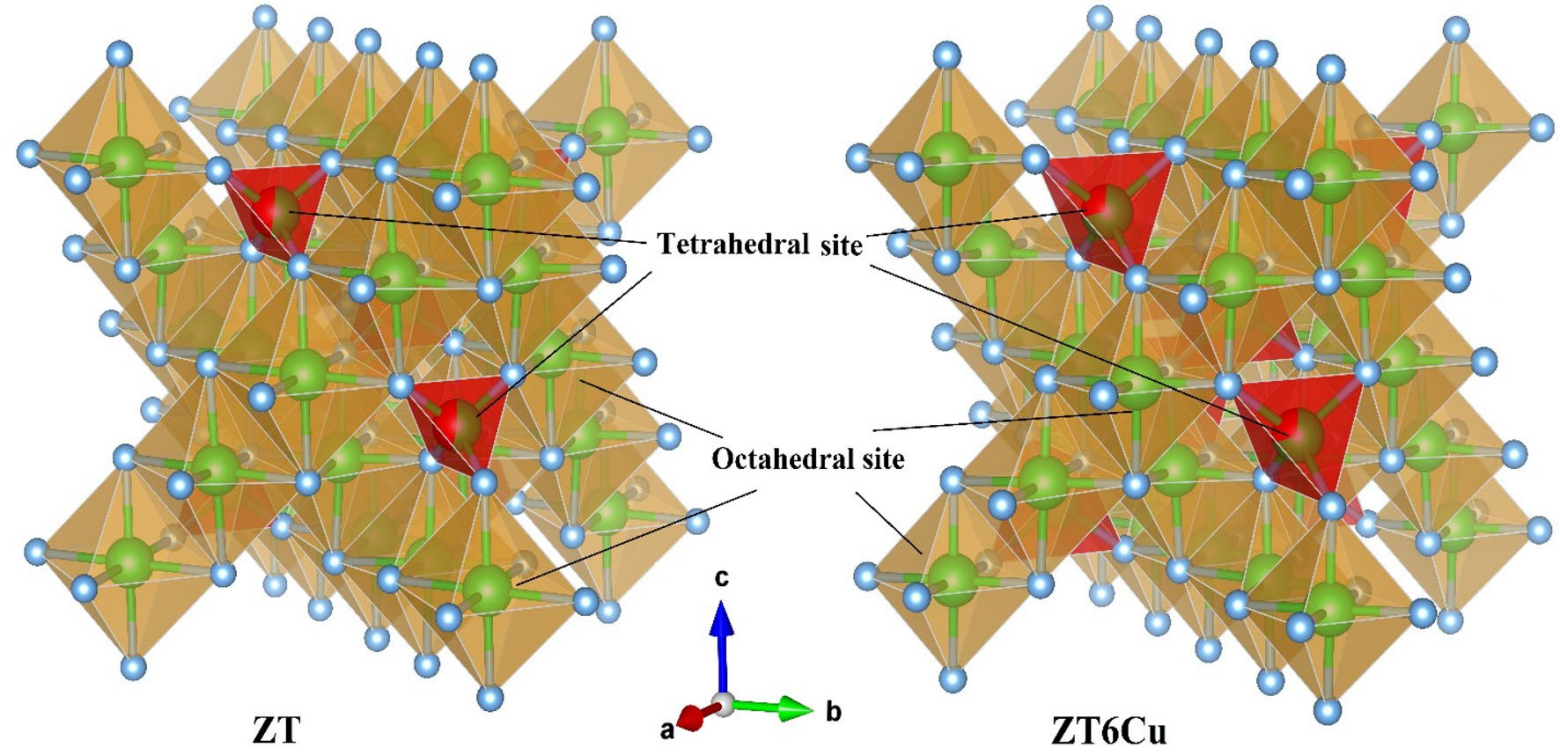
A schematic representation illustrating the crystal structure of Zn_1.6_Na_0.4_TiO_4_ and Zn_0.4_Na_0.4_Cu_1.2_TiO_4_.

Figure 4 shows a schematic representation for the ionic distribution in the crystal structure according to the Rietveld refinement data. The tetrahedral sites were shared between Ti^4+^ and (Zn^2+^, Na^+^, and Cu^2+^) ions, while the octahedral sites were occupied with (Zn^2+^, Na^+^, and Cu^2+^). The occupancies change can be returned to the effect of Na and Cu ions^56,57^.

The crystallite size of the fabricated samples was from the most intense peak calculated according to the Scherrer equation:

$$G= \frac{k\lambda }{\beta cos\theta }$$

In this context, the parameter 'k' is set to 0.9, representing the correction factor. 'β' represents the full width at half maximum of the chosen peak, and 'λ' denotes the XRD wavelength, which is 1.5406 Å.

The theoretical density (D_th_) was calculated from the following formula:

$${D}_{th}= \frac{8 {\text{Mw}}}{{N}_{a}{a}^{3}}$$

In this context, Na is the Avogadro’s number, a^3^ is the unit cell volume, and Mw is the molecular weight of the compound and it given by

$${\text{Mw}} = \left( {{1}.{6} - {\text{x}}} \right)*{\text{ Mw}}_{{{\text{Zn}}}} + 0.{4}*{\text{Mw}}_{{{\text{Na}}}} + {\text{x }}*{\text{Mw}}_{{{\text{Cu}}}} + {\text{Mw}}_{{{\text{Ti}}}} + {4}*{\text{Mw}}_{{\text{O}}}$$

The crystallite size with the theoretical density can be used to obtain the specific surface area (S_A_) according to the following formula

$${S}_{A}= \frac{6000}{{D}_{th}*G}$$

The substitution of Zn ions with Cu ions leads to a change in the lattice parameters of the prepared samples, which may, in turn, affect the electronic and optical properties, thereby influencing the sensor performance.

### Transmission electronic microscopy

**Figure 5:**
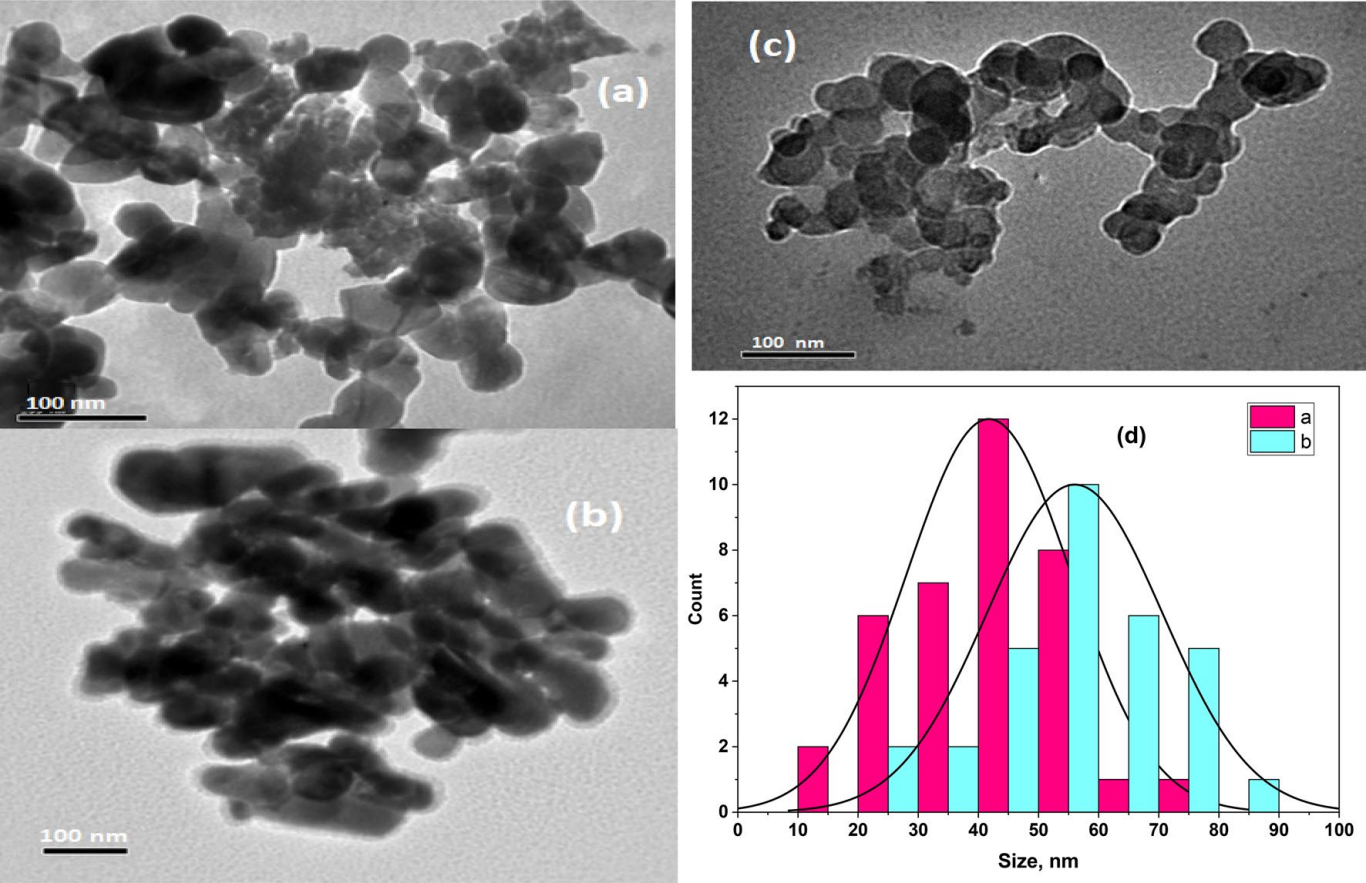
TEM images of (**a**) the Zn_1.6_Na_0.4_TiO_4_ spinel nanostructure, (**b**, **c**) the Cu-doped (0.6 Cu), and (**d**) the distribution of nanoparticles (loaded as a Supplementary file).

In order to comprehend the impact of Cu additive-induced changes in the ZnNaTiO_4_ network, it is essential to explore the formation of the Zn_1.6_Na_0.4_TiO_4_ spinel and its Cu-doped counterpart. This investigation is crucial as internal rearrangements may influence the quality of the Zn_2_TiO_4_-based spinel nanostructures. Figure 5 presents TEM images of both the Zn_1.6_Na_0.4_TiO_4_ and Cu-doped (0.3 and 0.6 Cu) spinel nanostructures (Fig. 5a–c). Both the pure and doped samples exhibit regular hexagonal-shaped nanoparticles with good distribution and cohesive clustering, showcasing distinct grains. In Fig. 5d of the nanoparticles distribution, it is evident that the Zn_1.6_Na_0.4_TiO_4_ spinel possesses an average grain size of 36–45 nm, whereas the Cu-doped (0.3, 0.6 Cu) spinel exhibits a slightly larger average grain size of 55 nm. This outcome can be attributed to the fact that the Cu cations, when substituting for the Zn ions, have a slightly smaller ionic radius. Consequently, the incorporation of these smaller cations into the ZnNaTiO_4_ lattice leads to a change in its lattice size^11^.

As depicted in Fig. 5d, the Zn_1.6_Na_0.4_TiO_4_ spinel exhibits an average grain size of 45 nm, while the Cu-doped (0.6 Cu) variant has a larger average grain size of 55 nm. This divergence in grain size suggests that distinct properties in terms of morphology and the distribution of nano-hexagonal particles arise due to the influence of varying levels of Na and Cu in the development of the Zn_2_TiO_4_ nanostructure. Furthermore, the diameter range of the resulting spinel nanostructures has shifted from 40 to 60 nm.

This variation in nanoparticle size, which includes both spherical and hexagonal particles, enhances the applicability of humidity sensors. This enhancement is attributed to their heightened adsorption properties, as previously noted by Ashok et al. and Chun et al.^16,58,59^.

### Raman study

**Figure 6:**
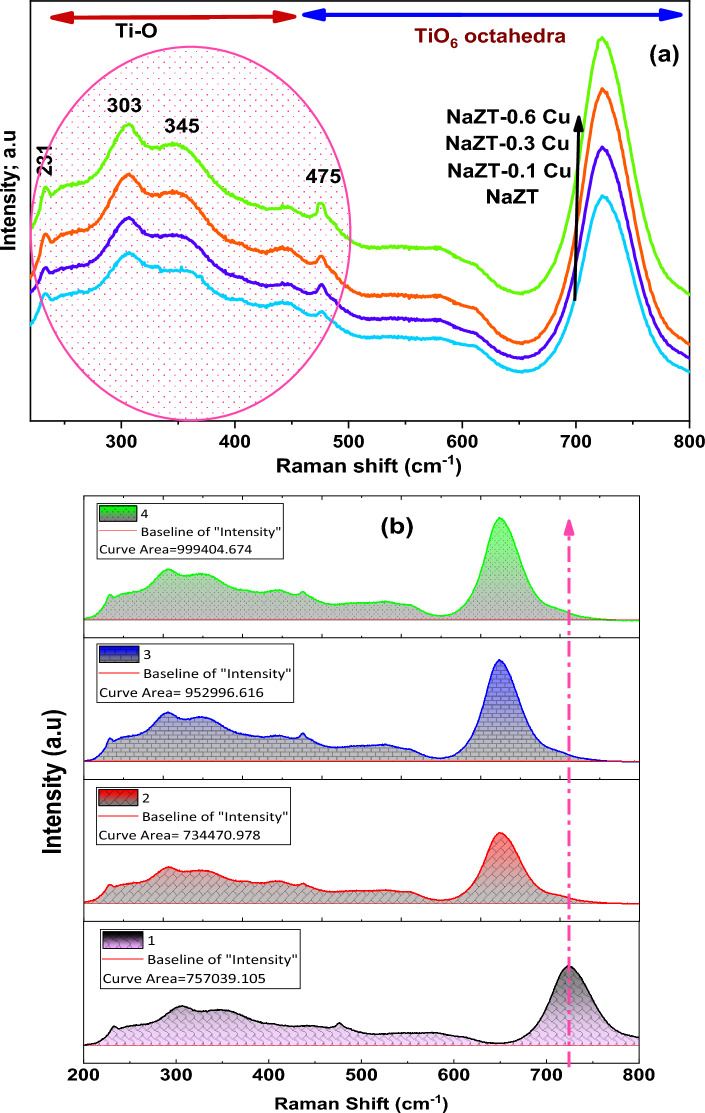
Raman spectra of (**a**) NaZT spinel nano-structure doped with different ratios (0.1–0.6 mol.%) of Cu NPs, and (**b**) the area under curve for the Raman spectra, calcined at 700 °C.

Raman spectroscopy proves to be a crucial instrument for investigating the dopant placement within nanoceramic systems. By analyzing the frequency and consistency of Raman vibrational modes, it assesses the polarizability modes and detects deviations in Raman spectra caused by doping divergences^60,61^. Functional Zn_(1.6 − x)_Na_0.4_Cu_x_TiO_4_ spinel nanostructures information was analyzed by Raman spectroscopy, the Raman spectra are shown in Fig. 6. Examining the structural alterations and the consequent shifts in Raman spectra in Cu NPs-doped NaZT spinel nanostructures, one could roughly infer that these changes predominantly arise from modifications in angles within the perovskite structure and variations in Zn–O and Ti–O distances.

As depicted in Fig. 6, the Raman spectra exhibit substantial alterations under the influence of the applied electric field. The introduction of Cu notably increases the intensities of Raman modes at the 233 cm^−1^ and 475 cm^−1^ frequencies, as well as the shoulder at 345 cm^−1^, while there is a slight increase in intensity around the 303 cm^−1^ modes^61–63^. The formation of the NaZT nanostructure was validated by the emergence of Raman vibration modes involving Ti–O–Ti and Zn–O within the spectral range of 200–800 cm^−1^.

The change in the Raman mode intensities (233, 345, and 475 cm^−1^) was ascribed to alterations in the level of disorder within the Ti–O and TiO_6_-octahedral structure of the NaZT matrix^64,65^.

The Raman vibrational modes of NaZT were identified at 231, 262, 348, 303, 345, and 475 cm^−1^, corresponding to the Eg, Ag, Eg, Eg, and Ag symmetrical Raman modes, respectively^60,66^. The Raman mode at 475 cm^−1^ displays a marginal correlation with the concentration of Cu, as depicted in the inset of Fig. 6a. With an increase in Cu concentration, the peak intensity rises, accompanied by a broadening of the peak width.

Hence, it is widely accepted that Raman modes within various frequency ranges are associated with lattice vibrations occurring in distinct regions of the perovskite framework^61^. Figure 6b depicting the Raman results for the area under peak, illustrates the alterations in the area under the peaks in the spectra of NaZT and Cu doped spinel nanostructure. These changes are primarily attributed to modifications within the internal network of the NaZT structure and occur within the 200–800 cm^−1^ range.

### Optical studies

**Figure 7:**
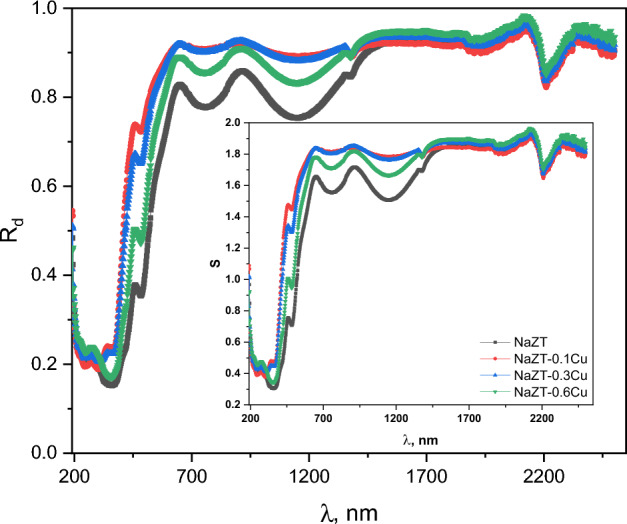
The diffuse reflectance of the prepared NaZnTiO_4_ dopped by Cu nanoparticle samples as a function of wavelength.

The diffuse reflectance (R_d_) measurements of the NaZT doped with copper nanoparticles were carried out in the wavelength span of 190–2500 nm. Figure 7 depicts the diffuse reflectance of the prepared nanoparticle samples as a function of wavelength. The inset of Fig. 7 shows the scattering (S) behavior with increasing wavelength, where it tacks the same form of the reflectance. Both R_d_ and S have the lowest value at low wavelengths below 350 nm. At 350 nm, R_d_ and S begin to increase to reach the saturation maximum state at about 650 nm.

**Figure 8:**
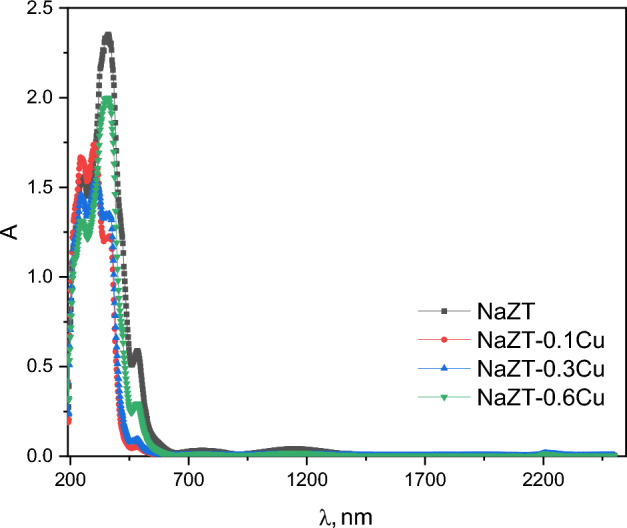
The optical absorption spectra of NaZnTiO_4_ doped with copper nanoparticles from 190–2500 nm.

The optical absorption spectra of NaZnTiO_4_ doped with copper nanoparticles from 190 to 2500 nm (Fig. 8) were obtained from reflectance values using Kubelka–Munk (K-M) function^24,68^, F(R) = (1 − R)^2^/2R, where R is the reflectance. The absorbance is expected to depend on several factors, such as band gap, oxygen deficiency, roughness, and impurity centers^69–71^. The prepared samples have strong absorbance in the ultraviolet region (lower wavelength) of the spectrum and a low absorbance in the higher wavelengths. The absorbance spectra show absorption peaks at about 250, 358, and 484 nm respectively.

The 1st peak is related to TiO^72–74^, where the charge carriers are electrons, whose density can be tuned by controlling oxygen deficiency via partially reducing Ti ions or partially substituting them with trivalent ions in the stoichiometric material (i.e., doping with oxygen vacancies). Akishige et al.^75^ have adopted the following picture of the band structure to interpret the transport property of their unstoichiometric BaTiO_3−δ_ materials. According to Akishige et al.^75^, the band gap is an increasing function of Ti–O bond distance and larger for a tetragonal BTO than for a hexagonal one. It is noted that the impurity states due to oxygen vacancy doping play the role of electron trapping centers, e.g., Ti^3+^, V_O−e_, or V_O−2e_, that produce donor levels in the band gap, which is ∆E in energy apart from the bottom of the conduction band^76^. The 2nd peak is related to ZnO^77–79^, where it is attributed to the low rate of recombination of the excited electron–hole pairs of Zn_2_TiO_4_ NPs with the impact on higher photo-adsorption of O_2_^78^. This in turn leads to the chance of producing a self-cleaning feature^78^. The noted enhancement of absorbance proposed a more UV–Vis photocatalytic reactivity^78^. Also, El Nahrawy et al.^55^ found a strong absorption peak at about 300 nm for Zinc Magnesium Titanate which is attributed to the photoexcitation of electrons from the valence to the conduction band and associated with the Zn_2_TiO_4_ phase. The 3ed peak is related to Na^80–82^ ions, where it originates from an electron transition between NaO oxygen 2*p* and Cu transition metal ion 3*d* states^83,84^. Achieved stronger UV absorption due to the high refractive index of TiO_2_ particles, improving UV-shielding of the device. TiO_2_ aggregation reduces light-scattering efficiency and decreases UV absorption.

The intensity of absorption decreases abruptly as the concentration of Cu increases to 0.01 but it starts to increase with further increasing of Cu doping. The decreasing absorption by initial doping of Cu may be due to the increase of the density of the defects, which results from the differences in size between dopant and host material^85^. The further increase of Cu which leads to the increase of absorption may be due to the removal of defects and disorders present in the sample^86^.

**Figure 9:**
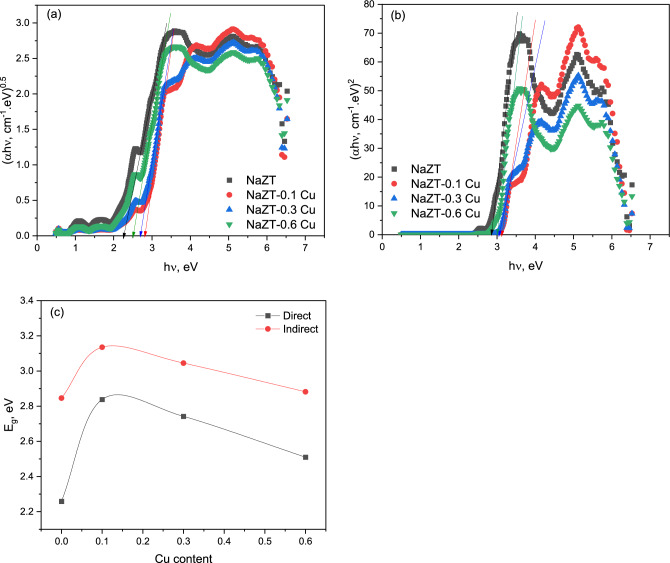
The Kubelka–Munk plots for the prepared samples regarding (**a**) direct and (**b**) indirect transitions. (**c**) the change of band gap energy with Cu addition.

Optical bandgap values for the prepared samples were calculated through the Kubelka–Munk (K–M) treatment. Figure 9a,b) illustrates the Kubelka–Munk plots for the prepared samples regarding direct and indirect transitions, which were used to calculate the optical band gap energies. The magnitude of the band gap (direct and indirect) increases with the addition of Cu and then decreases for further addition as shown in Fig. 9c. The initial increase of band gap with the addition of Cu may be due to the increase of the density of the defects which results from the differences in size between the dopant and host material^85^. With the further addition of Cu, a decrease in band gap is detected and may be due to the removal of defects and disorders^86^. From Fig. 9c, it is observed that the values of direct band transitions need a smaller energy than the indirect band transition and so it is more probable than the indirect type.

### Humidity sensors: sensor testing and evaluation

A closed conical flask contained a saturated salt solution, which was employed to establish specific humidity levels. Various saturated salt solutions, including LiCl, K_2_CO_3_, NaBr, NaCl, KCl, and K_2_SO_4_, were utilized to generate humidity levels of 11%, 23%, 43%, 57%, 84%, and 97%, respectively. Before the actual testing, the sensor was exposed to both low and high humidity levels for a duration of 24 h. The changes in impedance in relation to humidity levels were measured using an LCR bridge (HIOKI-3538-50) within the frequency range of 200–100 kHz. All measurements were conducted at room temperature.

**Figure 10:**
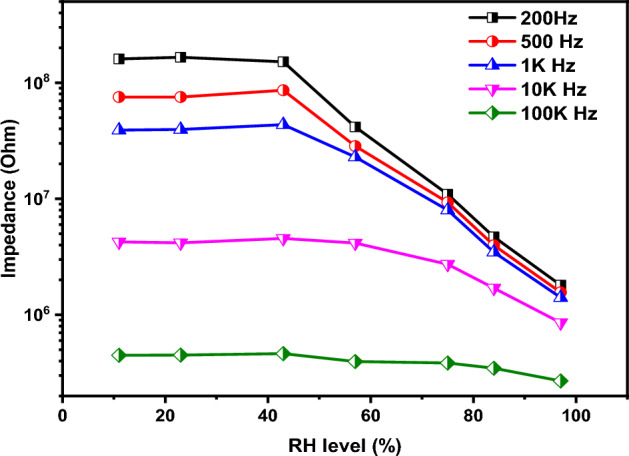
The impedance variation as a function of humidity at different testing frequency.

The primary determinant of the humidity sensor's response is the testing frequency, as illustrated in Fig. 10. The sensor was exposure to various humidity levels ranging from 11 to 97%, while subjected to a 1 VAC stimulus. The figure reveals that the impedance variation diminishes with increasing testing frequency. This observation aligns with the concept of water molecule polarizability. As the frequency rises, water molecules are less able to keep pace with the rapid frequency changes. The peak impedance variation occurred at 200 Hz, leading to the selection of this frequency for subsequent testing. Additionally, the sensor demonstrates a very little variation in its impedance when subjected to humidity levels up to 43%, then a linear and sharp decrease in its impedance was observed. The hysteresis due to humidification and de-humidification is one of the most key parameters that deserve to be studied. The hysteresis value can be determined using the following Eq. ^87,88^:

1 $$H= \frac{{Z}_{D}-{Z}_{A}}{S}$$

where ZD and ZA are the impedance values of the sensor at specific humidity level measured during the adsorption and desorption respectively.

**Figure 11:**
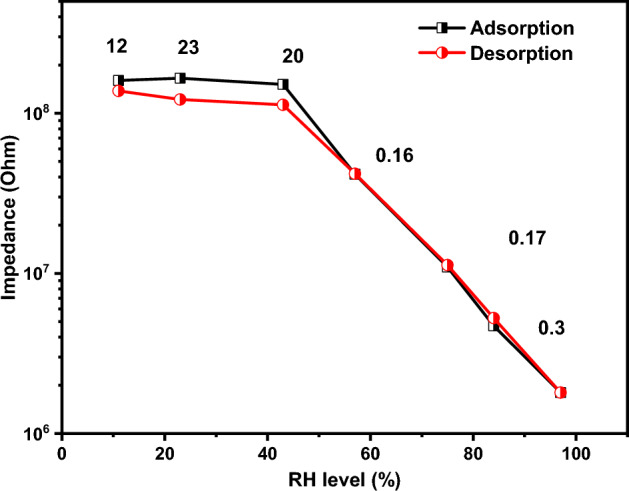
The hysteresis of the fabricated sensor.

**Figure 12:**
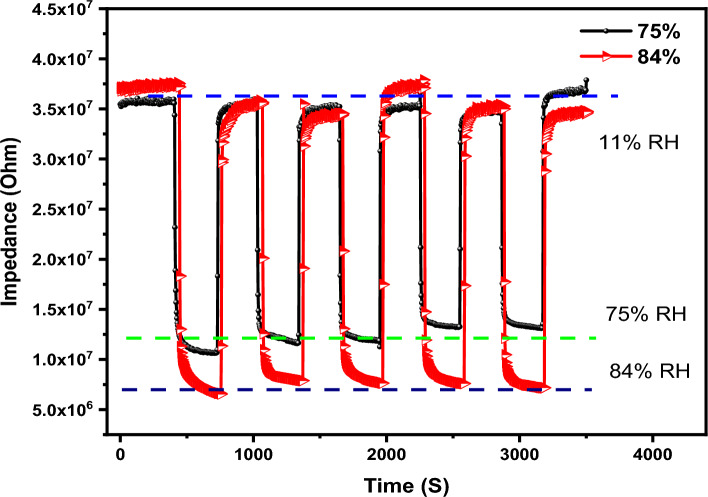
The repeatability of the fabricated sensor as a function of time at two different humidity levels.

Figure 11 illustrates the hysteresis of the examined sensor. At lower humidity levels, the hysteresis exhibited elevated values, reaching a peak of 23. The behavior of the sensor was changed as the humidity level increased. The highest recorded hysteresis value of 0.17 was observed at 57% humidity, suggesting the sensor's potential suitability for high humidity applications. Further characterization of the tested sample includes evaluation of its response and recovery properties. The response and recovery at different humidity levels as a function of time is illustrated in Fig. 12. The sensor exhibits excellent reversibility when transitioning between two distinct humidity levels. Additionally, it demonstrated minimal drift, showcasing its suitability for various applications.

**Figure 13:**
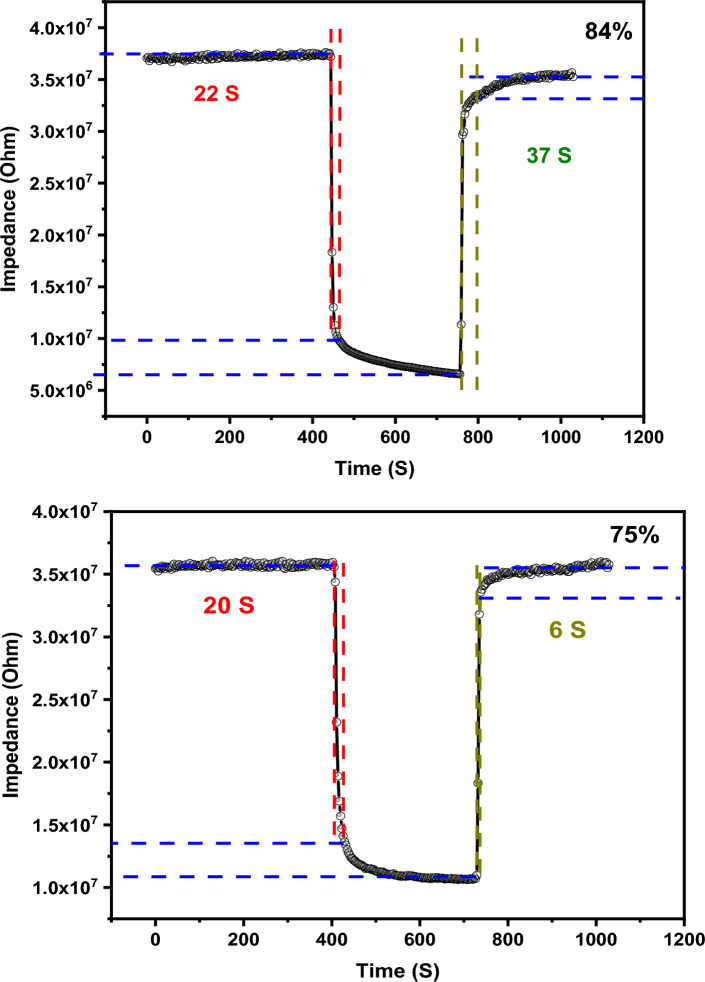
The response and recovery times of fabricated sensors at different humidity levels.

The response and recovery times were also studied to examine how fast sensors can respond to the humidity variation as shown in Fig. 13. Response time is the time required by the sensor to attain 90% of its maximum value. While the recovery time refers to the exerted time by the sensor to reach back to 90% of initial (baseline) value. The response and recovery times of the studied sensors were measured for two different humidity levels 75% and 84%. The calculated response and recovery times were found to be 20 s and 6 s when the humidity changed from 11 to 75%, respectively. The response and recovery times of the sensor were estimated to be 22 s and 37 s when the humidity changed from 11 to 84%. It was obvious that the fabricated sensor experienced a fast response and recovery in just seconds. The fast reversibility in addition to low hysteresis nominate this sensor to be applied in such applications that require smart humidity sensor.

**Figure 14:**
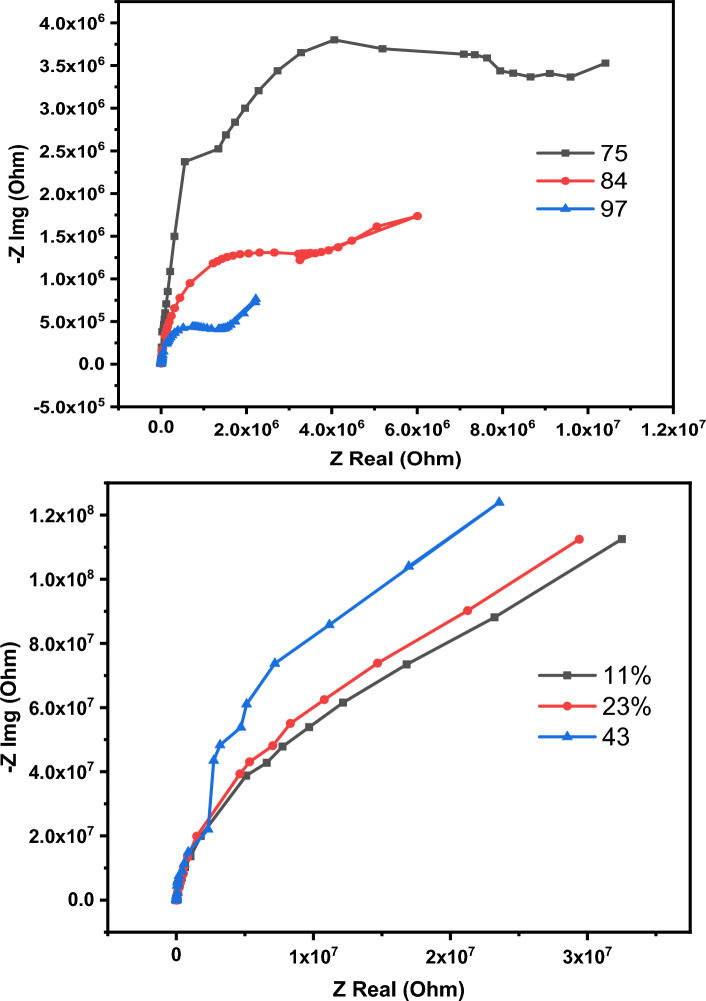
The Cole–Cole diagram of humidity sensor at different humidity levels.

The sensing mechanism of humidity sensor can be explained based on complex impedance spectroscopy (CIS) measurements. In such measurements the sensor is subjected to a specific level of humidity then the real part and imaginary part of impedance recorded. The CIS curves of the sensor at different humidity levels are illustrated in Fig. 14. These curves can broadly divide into main categories, at low and high humidity levels. In the low humidity level up to 43% a straight line is developed as the humidity increases, a semicircle connected to straight line starts to appear. The CIS curve's shape and curvature play a pivotal role in defining the charge transfer and interactions between surface-bound water molecules and the sensor's surface. These water molecules can be envisioned as forming sequential adsorbed layers. In conditions of low humidity levels, when a water molecule makes contact with the sensor's surface, a double hydrogen bond forms between the adsorbed water molecules and the sensor's surface. In this scenario, charges become localized and cannot move freely, resulting in a restricted impedance change. In such cases the adsorbed water molecules interact with the active sites of the sensor to generate hydroxyl ions. As the humidity increases, a second layer of water molecules are adsorbed physically. This stage is accompanied by the generation of mobile hydronium. These hydroniums in turn interact with hydroxyl ions to produce more hydronium. This reaction is continuous and known as chain reaction or Grotthuss reaction. The full mechanism can be described by the subsequent equations:

2 $${\text{H}}_{{2}} {\text{O}} \to {\text{H}}^{ + } + {\text{ OH}}^{ - }$$

3 $${\text{H}}_{{2}} {\text{O }} + {\text{ H}}^{ + } \to {\text{H}}_{{3}} {\text{O}}^{ + }$$

4 $${\text{H}}_{{3}} {\text{O}}^{ + } + {\text{ H}}_{{2}} {\text{O}} \to {\text{H}}_{{3}} {\text{O}}^{ + } + {\text{ H}}_{{2}} {\text{O}}$$

The humidity sensing outcomes were juxtaposed with those of previously reported sensors utilizing metal oxides, as illustrated in Table 2.

**Table 2:** Humidity sensing results from literatures compared with Zn_(1.6 − x)_Na_0.4_Cu_x_TiO_4_ nanostructures.

Sensing material	Fabrication technique	Range (RH%)	Response time (s)	Recovery time (s)	References
Cu_0.5_Zn_0.5_Fe_2_O_4_	Sol–gel self-combustion	11–98	350	N/A	^89^
ZnAl_2_O_4_	Co-precipitation	11–95	15	30	^90^
CuMn_2_O_4_	Low temperature stirring	22–94	20	47	^91^
MgCr_2_O_4_–TiO_2_	Citrate–nitrate gel	0–100	4200	5400	^92^
Mn_3.15_Co_0.3_Ni_0.8_O_4_	Sol–gel technique	12–95	30	50	^93^
ZnNa_0.4_Cu_0.6_TiO_4_	Sol gel	11–97	22	37	This work

## Conclusion

Hexagonal and spherical nanostructures of Zn_1.6_Na_0.4_TiO_4_ and Cu-doped spinel were effectively synthesized through a meticulously controlled sol–gel approach followed by a calcination process at 700 °C. The XRD analysis reveals that the spinel nanostructures possess a cubic crystal structure (Fd-3 m), and this structure's lattice parameter is affected by the Cu ratio. Additionally, the Rietveld refinement data demonstrates that the tetrahedral sites within the structure are jointly occupied by Ti^4+^ ions and (Zn^2+^, Na^+^, and Cu^2+^) ions, while the octahedral sites are filled with (Zn^2+^, Na^+^, and Cu^2+^) ions. The TEM reveals that both the Zn_1.6_Na_0.4_TiO_4_ and Cu-doped spinel nanostructures are characterized by the presence of nanoparticles displaying uniform hexagonal and spherical shapes with average diameters from 40 to 60 nm. This uniformity and control over nanoparticle morphology and size are promising attributes for potential applications in various fields, including sensor technology, sensors, and materials science. The both types of band gap (direct and indirect) increase on addition of Cu and then decreases for further addition. The Cu ratio had a discernible impact on the Raman-active modes of the Zn_1.6_Na_0.4_TiO_4_ spinel nanostructure, and these effects were effectively characterized through Raman scattering. The change in Raman modes upon the introduction of Cu NPs into the spinel nanostructures signifies the elevated crystallinity of the spinal nanostructures, underscoring their efficacy in humidity sensing applications. Hence, the initial rise is linked to the heightened density of defects arising from the disparities in size between the dopant and the host material. The later decrease of band gap is related to the removal of defects and disorders. The direct transition type is more probable than indirect type. The findings underscore the suitability of these materials for precise and controlled nanostructure fabrication. The humidity sensing performance of the Zn_(1.6 − x)_Na_0.4_Cu_x_TiO_4_ spinel nanostructure was assessed across a wide humidity range at various testing frequencies. The highest sensitivity was achieved at 200 Hz. Notably, this sensor displayed a remarkable ability to respond to changes in humidity, with response and recovery times measured at 20 s and 6 s, respectively. The porous nature of these samples led to accelerated water absorption kinetics, as evidenced by the rapid change in conductivity with shifting humidity levels. This suggests the potential for developing an innovative conductimetric sensor for precise humidity measurement. Given its exceptional humidity sensing capabilities, the Zn_(1.6 − x)_Na_0.4_Cu_x_TiO_4_ spinel nanostructure humidity sensor holds significant promise for a range of applications, including monitoring human respiration rates, environmental sensing, non-contact finger sensing, and many others.

### Supplementary Information

Supplementary Information 1.

Supplementary Information 2.

Supplementary Information 3.

Supplementary Information 4.

Supplementary Information 5.

Supplementary Information 6.

Supplementary Information 7.

Supplementary Information 8.

Supplementary Information 9.

## Data Availability

All data generated or analyzed during this study are included in this published article [and its supplementary information files].
